# Dental fear and perceived oral health importance: A mediation analysis of courage as virtue

**DOI:** 10.6026/973206300210231

**Published:** 2025-02-28

**Authors:** Supriya S, Amra Ahsan, Rajbir Singh

**Affiliations:** 1 Department of psychology, SGT University, Gurugram, Haryana, India

**Keywords:** Courage, dental fear, health behaviour, oral health behaviour, periodontitis

## Abstract

The impact of dental fear on the perceived importance of oral health is of interest to dentists. Hence, 100 participants with clinical diagnoses of dental caries and periodontal/gingival diseases were assessed. The dental fear survey comprised 20 items in the questionnaire addressing the different situations, associated feelings and reactions related to dental treatment. Perceived importance of oral health was recorded through 3 items of oral health belief questionnaire. Character strengths of courage were recorded through the Values in Action Inventory of Strength (VIA-IS) Questionnaire (40 items; 10 items belonging to each of the four character strengths of courage). Later, a mediation analysis was conducted to evaluate the direct effect of dental fear and its domains, as well as the mediating effect of courage and its domains (perseverance, honesty, zest, bravery) on perceived importance. The 'physiological arousal' and 'avoidance of dentistry' factors associated with dental fear negatively impact the perceived importance of oral health. Thus, virtue of courage has the potential to improve the perceived importance of oral health.

## Background:

Confronting individual fears is not an uncommon finding during various situations in our lives. The virtue that enables us to do so effectively is courage. Virtue of courage is required to overcome our fears of associated perceived risks in an action for a positive outcome. It is an essential ingredient of the model of character proposed by Peterson and Seligman [1]. Human functioning is a reflection of psychological functioning. Trends of research in exploring and extracting psychological strengths to improve health outcomes are gaining momentum [2, 3- 4]. The burden of chronic diseases with modifiable risk factors is increasing with trends of increasing longevity [5]. Oral diseases are not an exception among them. The significance of oral health is not restricted to masticatory efficiency, phonetics and aesthetics of the facial region. Risk factors for rheumatoid arthritis, cardiovascular diseases, respiratory diseases, mood disorders, eating disorders and depression have been linked to oral diseases [6, 7]. Fear and anxiety associated with the invasive nature of dental treatment are not an uncommon finding across cultures and populations [8, 9]. It has been reported to be one of the most common reasons for non-compliance or erratic compliance to dental appointments [10, 11]. Regular compliance is a vital link to the long-term management of periodontal diseases [12]. Extracting and utilising individual character strengths of courage may be among the potent means for overcoming this psychological barrier to oral health. Findings of a recent study also hint towards the probability of the impact of modulating factors when fear of dentistry is considered regarding oral health behaviour [13]. Identifying mediators and moderators in behavioural intervention strategy is important for its efficient applications in bringing health behaviour changes. Mediators are linked to the processes belonging to the functioning of an intervention. Psychosocial variables, including intrinsic motivation and self-efficacy, may be responsible for influencing the outcomes of an intervention meant for health behaviour modifications. Moderators reflect on circumstances influencing the efficacy of an intervention. Intrinsic motivation refers to engaging in an activity of pleasure. The activity being a direct source of pleasure is vital for intrinsic motivation. If the outcome of the activity is an indirect cause of engagement, it is extrinsic motivation. Positive oral health behaviours (facing invasive dental procedures) are less likely to be driven by intrinsic motivation. However, the scope of extrinsic motivation being internalized cannot be ruled out [14]. Internalization means long-term stable behaviours or autonomously regulated behaviours. Long-term stability is important in health behaviours. The impact of dental anxiety over the perceived importance of oral health and the modulating potential of character strengths of virtue of courage may be an interesting context to be investigated in devising effective strategies for the internalization of extrinsic motivation for positive oral health behaviours. The dental fear survey has been used in behavioural research. It has been reported to exhibit good stability and acceptable validity across cultures [15]. Individual beliefs about the seriousness of the disease and its importance may act as an important determinant of health behaviour. Hence, items about the theoretical construct of the perceived importance of oral health belonging to the oral health belief questionnaire were considered in the present study [16]. The perspective on human behaviour under consideration is important. Positive psychology advocates the employment of character strengths for optimum human functioning. Values in Action Inventory of Strengths (VIA-IS), developed by Peterson and Seligman (2004), measures 24 character strengths through 240 items [1]. Therefore, it is of interest to assess dental fear and perceived oral health importance using a mediation analysis of courage as virtue.

## Methods and Materials:

## Study design and study population:

The ethics clearance certificate was obtained from the Ethical Committee of the Faculty of Behavioural Sciences, Shree Guru Gobind Singh Tricentenary University, Gurugram, Haryana, for this cross-sectional study. The study was conducted with ethical guidelines as per the Declaration of Helsinki 1975 (2013). 100 patients, 20-40 years old, diagnosed clinically with tooth decay (dental caries) and/or gum diseases (gingivitis, periodontitis) were enrolled from the Postgraduate Institute of Dental Sciences. The sample size was determined by using the formula N= 4 PQ/d2. Where P is the prevalence of oral diseases in percentage (55.2%), Q is 100-P when P is in percentage (100-55.2=44.8). Relative precision as a proportion of P is the value of d in the formula. The relative precision of 18% was considered in the 55.2% prevalence of oral diseases to calculate a sample of 100 participants. (4x55.2x44.8)/9.936x9.936= 100.19.

## Eligibility criteria:

Patients diagnosed clinically with gum diseases/decay of teeth (gingivitis, periodontitis, or dental caries) were enrolled. Written informed consent was obtained. History of psychiatric disorder was the exclusion criterion in this study. The putative interactions between character strengths of courage and the perceived importance of oral health in the background of dental fear were investigated by employing the following tools. English versions of the tools were applied to the participants. The participants were assisted by researchers to understand the contents of items of tools administered if required.

## Tools:

## Dental fear survey (DFS):

DFS comprised 12 items concerning fear of specific stimuli, five items of physiological arousal, two items belonging to 'avoidance of dentistry' and 1 item regarding overall dental fear. 15 Each item was rated on a 5-point Likert scale, ranging from 1 (least rating of emotion of dental anxiety and fear) to 5 (highest rating of emotion of dental anxiety and fear).

## Oral health belief questionnaire (OHBQ):

OHBQ, comprising 18 items, measured the perceived importance of oral health, perceived seriousness of oral diseases, perceived barriers to oral health care and efficacy of dentists along with benefits of oral hygiene practices. 16 items regarding theoretical construction of perceived importance of oral health were considered for the present study. Items were scored on a dichotomous scale and added to a sum for each item. The sum represented the score for the perceived importance construct of OHB. The three items considered for evaluating this dimension of oral health belief are: I place great value on my dental health, it is important to keep natural teeth, and dental disease is as important as other health problems.

## Values in action inventory of strength (via-is) questionnaire:

VIA-IS, developed by Peterson and Seligman (2004), measure 24 character strengths through 240 items [1]. Each item is scored ranging from the lowest score of 1 (very much unlike me) to the maximum score of 5 (very much like me). The present study was undertaken to analyse the mediating potential of character strengths accompanying the virtue of courage. Hence, the four associated character strengths that constitute courage were considered in the present study.

## Virtue of courage includes four character strengths:

[1] Bravery (valour)

[2] Perseverance (persistence)

[3] Integrity (honesty)

[4] Zest (enthusiasm, vitality, vigour)

## Statistical analysis:

In this study, mediational analysis was conducted to evaluate the direct effect (D.E.) of dental fear and its domains, as well as the mediating effect of courage and its domains (perseverance, zest, bravery) on perceived importance. The software JAMOVI (Version 2.3.28) was accessed on 7th March 2024 to test the mediational hypotheses.

## Results:

Character strengths of bravery, perseverance, zest and honesty were evaluated for their impact on the perceived importance of oral health. The mean age of the study population was 29.370 ± 6.426 years. Females and males had statistically similar mean ages of 28.760 ± 6.176 years and 29.980 ± 6.674 years, respectively (P =.371). The mean perceived importance in the study population is 2.880 ± .326. Females and males had mean perceived importance of 2.840 ± .370 and 2.920 ± .274 respectively (P =.221). The mean and median values of physiological arousal in the study population are 9.840 ± 4.811 and 8.0, respectively. Females and males had mean physiological arousal of 10.940 ±4.904 and 8.740 ±4.498, respectively, with median values of 10.000 and 7.000 (P =.013). The mean dental fear total in the study population is 41.430 ± 18.794. Females and males had a mean dental fear total of 45.860 ± 17.512 and 37.000 ± 19.155. The median dental fear total in the study population was 39.000. Females and males had a median dental fear total of 44.000 and 29.500respectively. Females had statistically significantly higher dental fear total than males (P =.006). The mean bravery in the study population is 3.817 ± .534. Females and males had mean bravery of 3.762 ± .624 and 3.872 ± .426 respectively. The median bravery in the study population was 3.800. The mean perseverance in the study population is 3.939 ± .497. Females and males had mean and median perseverance of 3.940 ± .533 and 3.938 ± .463 and 3.900 and 3.950, respectively. It was statistically nonsignificant (P = .984). The mean zest in study population is 4.043 ± .448; 4.064 ± .461 (females) and 4.022 ± .439 (males) (Table 1). The median zest in the study population was 4.050. Females and males had median zest of 4.100 and 3.950 respectively (P =.642). The mean courage in the study population is 3.937 ± .424 (Table 1).

## Avoidance of dentistry:

In the first path analysis, avoidance of dentistry was considered as an independent variable, with perseverance and perceived importance as mediating variables and dependent variables, respectively. Avoidance of dentistry had a significantly negative effect on perceived importance (β = -.010, P =.034). Avoidance of dentistry had no significant effect on perseverance (β = .002, P =.766) and perseverance had a significant positive effect on perceived importance (β = .142, P =.024). Perseverance had no significant effect on the association between avoidance of dentistry and perceived importance (mediation = 3.07%, β = 3.08e-4, P =.768) (Table 2, Figure 1 - (see PDF)). There is a significant total negative effect on perceived importance (β = -.009, P =.045) (Table 2). The second path analysis consisted of avoidance of dentistry as an independent variable, zest as a mediator and perceived importance as a dependent variable (Figure 2 - see PDF). Avoidance of dentistry had a significant direct negative effect on perceived importance (β = -.010, P = .032) (Table 2). Avoidance of dentistry did not affect zest (β = .003, P = .636) (Figure 2 - see PDF).

Zest had a significant positive effect on perceived importance (β = .143, P = .041) (Figure 2 - see PDF). The indirect effect of avoidance of dentistry with zest as a mediator was nonsignificant (mediation = 4.33%, β = 4.47e-4, P = .644) (Table 2, Figure 2 - (see PDF)). The total effect on perceived importance was significant (β = -.009, P = .045)(Table 2). The third path analysis had avoidance of dentistry as an independent variable, courage as a mediator and perceived importance as a dependent variable (Figure 3 - see PDF). Avoidance of dentistry had a direct negative effect on perceived importance (β = -.010, P = .041) (Table 2, Figure 3 - (see PDF)). Avoidance of dentistry did not affect courage (β = 1.55e-5, P = .998) and courage had a significant positive effect on perceived importance (β =.146, P = .049) (Figure 3 - see PDF), with a non-significant indirect effect on perceived importance (mediation = .02%, β = -2.26e-6, P = .998) (Table 2, Figure 3 - (see PDF)). There was a significant total negative effect on perceived importance (β = -.010, P = .045) (Table 2).

## Physiological arousal:

In the fourth path analysis, physiological arousal was an independent variable; bravery was a mediator and perceived importance was a dependent variable (Figure 4 - see PDF). There was no significant direct effect of physiological arousal on perceived importance (β = -.012, P = .073) (Figure 4 - see PDF). Physiological arousal did not affect bravery (β = -.004, P = .741) (Figure 4 - see PDF). Bravery had a significant positive effect on perceived importance (β = .117, P = .048) (Figure 4 - see PDF). Bravery did not mediate the association between physiological arousal and perceived importance (mediation = 3.52%, β = -.429e-4, P =.744). The total effect on perceived importance in this model was non-significant (β = -.012, P =.068). In the fifth path analysis, physiological arousal and perseverance were independent variables and mediators, respectively, with perceived importance as a dependent variable (Figure 5 - see PDF). Physiological arousal had a direct negative effect on perceived importance. However, it was statistically non-significant (β = -.012, P =.069) (Figure 5A - see PDF). Physiological arousal did not affect perseverance (β = -.002, P = .817) (Figure 5 - see PDF) and perseverance had a significant positive effect on perceived importance (β = .135, P = .033) (Figure 5A - see PDF), with non-significant indirect effect (mediation = 2.65%, β = -3.22e-4, P =.818) (Figure 5A - see PDF). The total effect on perceived importance was statistically non-significant (β = -.012, P =.068).

The indirect effect of physiological arousal with zest as a mediator on perceived importance was non-significant (mediation = 4.05%, β = 5.37e-4, P = .690) (Figure 6 - see PDF). Physiological arousal had no significant effect on zest (β = .004, P = .684) (Figure 5B - see PDF). Zest had a significant positive effect on perceived importance (β = .142, P =.044). The total effect on perceived importance was non-significant (β = -.012, P =.068). In the seventh path analysis, physiological arousal and courage were independent variables and mediators, respectively, with perceived importance as a dependent variable (Figure 6A - see PDF). The direct effect of physiological arousal on perceived importance was non-significant (β = -.012, P = .066) (Figure 6A - see PDF). The physiological arousal had no significant effect on courage (β = -9.37e-4, P = .916) and courage had no significant effect on physiological arousal (β = .144, P = .052) (Figure 6A - see PDF), with non-significant indirect effect (mediation = 1.11%, β = -1.35e-4, P = .916) (Figure 6A - see PDF). The total effect on perceived importance was non-significant (β = -.012, P =.068).

In the eighth path analysis, dental fear was an independent variable, perseverance was a mediator and perceived importance was a dependent variable (Figure 6B - see PDF). Dental fear had a direct negative effect on perceived importance (β = -.004, P = .023) (Figure 6B - see PDF). Dental fear did not affect perseverance (β = .002, P = .533). However, perseverance had a significant positive effect on perceived importance (β = .147, P = 020) (Figure 6B - see PDF). The indirect effect of dental fear on perceived importance with perseverance as a mediator was non-significant (mediation =6.00%, β = 2.41e-4, P = .547) (Figure 6B - see PDF). The total effect of dental fear itself and mediated by courage was significant (β = -.004, P =.037). Mediation analysis of the 'avoidance of dentistry' factor of the Dental fear survey and perceived importance of oral health reveals that the virtue of courage and its character strengths of perseverance and zest have a significant impact on the perceived importance of oral health. Similarly, mediation analysis between the 'physiological arousal' factor of the dental fear survey and the perceived importance of oral health finds bravery, perseverance and zest character strengths of the virtue of courage impacting the perceived importance of oral health positively. Mediation analysis between dental fear and perceived importance of oral health, again, signifies the positive impact of perseverance character strength of virtue of courage over perceived importance of oral health.

## Discussion:

Facilitative or acceptable anxiety may influence the performance of positive health behaviours [17]. Hence, it may facilitate adopting preventive measures at the personal level as well as availing those at a professional level. However, dental anxiety, when increased beyond the critical range of healthy anxiety, may exert its pathological impacts [18]. Character strengths associated with the virtue of courage may act as a deterrent against emotions of anxiety and fear of dentistry, exerting its pathological impacts of maladaptive behaviours of non-compliance to oral health therapeutic recommendations. Perceived importance of oral health may be an important driving motivation factor to comply with primary preventive measures and adopt behaviours congruent with oral health [19, 20]. Data from population-based samples of adults participating in the ICS-II USA study was used to test the validity and reliability of oral health belief measures. All five scales comprising oral health beliefs were found to possess good validity and reliability. Perceived importance of oral health and perceived seriousness were highly correlated among the belief scales. The perceived importance of the oral health scale is used in the present study. Results of the present study reveal that avoidance of dentistry factor of dental fear is significantly impacting oral health beliefs regarding its 'perceived importance of dental health' construct. Mediating analysis reveals the mode of impact to be direct. The character strength of perseverance mediated the positive impact of avoidance of dentistry over perceived importance is observed to be insignificant. Path estimate reveals that this character strength of virtue of courage has a significant impact on the 'perceived importance of oral health' of individuals. However, as avoidance of dentistry factor of dental fear does not influence perseverance, the net mediated impact of this factor of dental fear becomes non-significant. The virtue of courage does not imply the absence of fear and anxiety [21]. It is the ability to act for positive outcomes despite the presence of fear of associated risks [22]. In the present study, items scored related to the anxiety of perceived or anticipated pain associated with the invasive nature of dentistry are a measure of perceived risk for undergoing dental treatment in the present study. Items related to avoidance of dentistry factor of DFS include putting off making dental appointment and non-compliance or erratic compliance to confirmed dental appointments. Compliance with therapeutic recommendations is vital to the long-term maintenance of oral health. However, poor compliance is routinely observed in chronic diseases perceived to be non-threatening, particularly in periodontal disorders. Promotion of compliance to oral health recommendations may be achieved through inculcating character strength of courage. It may not only be useful for attaining and maintaining oral health but also for the well-being of individuals. The findings of co-morbidity are dental anxiety with psychological disorders, including depression, further hint towards the role of character strength in the well-being of individuals. Identical patterns of mediation estimates and path estimates of interactions among 'avoidance of dentistry,' 'zest' and 'perceived importance of oral health' have been observed in our study. When the virtue of courage was considered to assess these interactions, the same trend of mediation impacts was observed between the virtue of courage and the 'perceived importance of oral health.' A comparable trend is also observed among interaction of the perceived importance of oral health with bravery and character strength. Another important factor of DFS is physiological arousal. The findings of the present study do not support that this factor of the dental fear survey has a significant direct or indirect impact on individuals' perceived importance of oral health. However, each of the character strengths associated with the virtue of courage, viz. perseverance, bravery and zest, reinforce the perceived importance of oral health in this path estimates again. Individual and community utilisation of health facilities for disease prevention is influenced by individuals and contextual factors. Perceived importance of the disease at individual and community level is important among these factors [19, 20].

Control of confounders in cross-sectional observational studies is important for the associations to be valid. Mediating and moderating variables may act as confounders. Positive psychology has popularised that a balanced view of individual appraisal requires the inclusion of individual resources and character strengths as well [23]. In a study to develop a grounded theory of courage among individuals with long-term health concerns, Finfgeld analysed qualitative data using grounded theory methods. Being responsible, productive and sensitive to significant others and the world in general is synonymous with courage [2]. Healthcare providers have the potential to facilitate this process through competence and effective communication. Outcomes of being courageous include personal integrity and thriving amid normality. In a study exploring the relationship between the frequency of health behaviours and the perceived importance of health behaviours, Nudelman and Ivanova observed in a population of 250 adult participants that the perceived importance of health behaviours and health behaviour performance were positively correlated [24]. They inferred that a model wherein importance affects performance, which in turn affects self-assessed health, was superior to a model wherein performance affects importance. They concluded that importance perceptions should be considered when developing behavioural interventions [24]. These findings are congruent with the results of the present study. In another investigation by Hsu and Chiang, the effects of BMI and the perceived importance of health on health behaviours (patterns of eating, sleeping and exercising) were explored among 334 college students. Interactions between BMI and perception of the importance of health for exercise behaviours (F2, 328=3.50, P=.03) were observed in this study. The authors concluded that the perception of health has a significant effect on exercise behaviours among college students [25]. These findings buttress the significance of the findings of the present study. Understanding mechanistic links explaining how health beliefs influence health behaviour may be relevant to improving individual preventative health behaviours. Data from a sample of approximately 60,000 respondents from 159 countries were used to explore associations between 24 character strengths (CS) and 15 health-related outcomes in a study by Bialowolska *et al.* [26] Character strengths yielding the most significant favourable associations across health-related quality of life outcomes were zest, self-regulation, hope and gratitude whereas those concerning health behaviours were zest and self-regulation. This study also supports the relevance of character strength zest under the virtue of courage in individual health behaviours and well-being [26]. Sasayama *et al.* observed that the physical fitness total score was related to perseverance-honesty, courage-ideas and compassion-gratitude [27].

The association of physical well-being with character strengths has been reported in previous studies. Proyer *et al.* thought that personality characteristics influence health by impacting compliance with health behaviours [28]. They believed that positive traits are related to preferred health behaviours in a meta-interpretation of six qualitative studies to understand the concept of courage among individuals experiencing a variety of threats to their well-being. Virtue of courage and its character strengths have been reported with their significant positive impact over dietary preferences congruent with oral health [29]. The scope of potential of these character strengths to modulate compliance to oral health behaviour has been suggested [29]. Key findings of the present study are: a) Mediation analysis of the 'avoidance of dentistry' factor of the Dental fear survey and perceived importance of oral health reveals that courage, along with the associated character strengths of perseverance and zest, have significant impact over perceived importance of oral health; b) similarly, mediation analysis between 'physiological arousal' factor of dental fear survey and perceived importance of oral health finds bravery, perseverance and zest character strengths of virtue of courage impacting perceived importance of oral health positively; c) mediation analysis between dental fear and perceived importance of oral health, again, signify positive impact of perseverance character strength of virtue of courage over perceived importance of oral health. The results of our study may help to broaden our understanding of interactions among emotions of dental fear, character strengths under the virtue of courage and the perceived importance of oral health. Though the study may be of little significance concerning its direct applications for dental health, it may provide background for further investigations in the field of the scope of character strengths of courage in the background of anxiety and fear for positive health behaviours and well-being. Much of the data in studies on emotions, virtues and perceptions are self-reported. This data is associated with biases such as social desirability bias, recall bias, or response bias. Participants might underreport their anxiety or over-report their courage due to social expectations.

Distortion effects due to probable bias associated with self-reported tools cannot be ruled out completely in the present study. The cross-sectional nature of the study allows the capture of data at a single point in time, which limits the ability to infer causality in the present study. Longitudinal studies are better suited to determine causal relationships. The study was conducted among patients attending dental health institutes for the management of their oral health problems. The external validity of the results of the study cannot be extended to the population at large. Within the limitations of a cross-sectional design in a relatively small population, the results regarding the association of potential mechanism of interactions between the perceived importance of oral health and character strengths belonging to the virtue of courage need to be taken with caution as they require further investigation and confirmation in multicenter longitudinal studies among diverse populations. The interactions and impact of other mediating variables, such as education about the disease, socio-economic status and other constructs of oral health beliefs, viz. perceived barriers, self-efficacy and a clue to action, need to be explored and ascertained in long-term interventional studies analyzing these impacts on oral health behaviour.

## Conclusion:

The 'physiological arousal' and 'avoidance of dentistry' factors associated with dental fear negatively impact the perceived importance of dental health. These results hint towards the relevance of perseverance, zest and bravery character strengths under the virtue of courage for impacting the perceived importance of oral health, an important construct of oral health beliefs. The mediating and moderating variables associated with this putative potential warrant further exploration in longitudinal studies for its applications in positive oral health behaviours.

## Supplementary material:

No

## Author Contribution:

Each author has made a substantial contribution to the conception or design of the work, acquisition, analysis and interpretation of data and has drafted the work and substantively revised it.

## Funding:

This research received no external funding.

## Institutional review board statement:

The study was conducted according to the guidelines of the Declaration of Helsinki, as revised in 2013 and approved by the Ethics Committee of Faculty of Behavioural Sciences, SGT University and Guru gram with protocol code SGTU/FBSC/ECC/2022/29.

## Informed Consent Statement:

Informed consent was obtained from all subjects involved in the study.

## Data Availability Statement:

The data presented in this study are available on request from the corresponding author. The data are not publicly available due to privacy reasons.

## Figures and Tables

**Table 1:** Comparison of demographic variable and 'perceived importance' domains of oral health belief, dental fear and courage

	**Variables**	**Total (n=100)**	**Females (n=50, 50%)**	**Males (n=50, 50%)**	**P value**
**Demographic variable**					
	Age (years)	29.370 ± 6.426	28.760 ± 6.176	29.980 ± 6.674	.371**
		29.000 (24.00-34.000)	29.000 (23.000-34.000)	29.000 (24.000-37.250)	
**Oral Health Belief**					
	Perceived importance	2.880 ± 0.326	2.840± 0.370	2.920± 0.274	.221**
		3.000 (3.000-3.000)	3.000 (3.000-3.000)	3.000 (3.000-3.000)	
**Dental fear**					
	Avoidance of dentistry	13.650 ± 6.803	14.940 ± 6.695	12.360 ± 6.730	.005*, **
		11.000 (8.000-17.000)	13.000 (9.750-18.250)	9.000 (8.000-15.000)	
	Physiological arousal	9.840 ± 4.811	10.940 ± 4.904	8.740 ± 4.498	.013*, **
		8.000 (5.000-13.000)	10.000 (6.000-15.250)	7.000 (5.000-11.250)	
**Courage**					
	Perseverance	3.939 ± .0497	3.940 ± 0.533	3.938 ± 0.463	.984***
		3.900 (3.600-4.300)	3.900 (3.575-4.325)	3.950 (3.600-4.300)	
	Honesty	3.952 ± 0.494	3.944 ± 0.541	3.960 ± 0.447	.872***
		4.000 (3.700-4.300)	4.000 (3.600-4.300)	3.900 (3.700-4.200)	
	Zest	4.043 ± 0.448	4.064 ± 0.461	4.022 ± 0.439	.642***
		4.050 (3.725-4.375)	4.100 (3.800-4.400)	3.950 (3.700-4.325)	
	Courage	3.937 ± 0.424	3.927 ± 0.471	3.948 ± 0.376	.811***
		3.875 (3.650-4.275)	3.875 (3.612-4.356)	3.900 (3.650-4.181)	
Data: Mean ± SD and (median),
*Statistical significance (P < .05),
**Mann-Whitney U test,
***Unpaired Student t test

**Table 2:** Effect of domains of dental fear on 'perceived significance of oral health' domain of oral health beliefs through domains of courage

	**Indirect Effect (IE)**					**Direct Effect (DE)**					**Total Effect**			
	**β**	**95% CI**		**% Mediation**	**P**	**β**	**95% CI**		**% Mediation**	**P**	**β**	**95% CI**		**P**
		**Lower**	**Upper**				**Lower**	**Upper**				**Lower**	**Upper**	
Avoidance of dentistry - Perseverance - Perceived importance	3.08E-04	-0.002	0.002	3.07%	0.77	-0.01	-0.019	-7.31E-04	96.93%	0.03	-0.009	-0.018	-2.01E-04	0.05
Avoidance of dentistry - Zest - Perceived importance	4.47E-04	-0.001	0.002	4.33%	0.64	-0.01	-0.019	-8.26 e-4	95.67%	0.03	-0.009	-0.019	-2.01E-04	0.05
Avoidance of dentistry - Courage - Perceived importance	2.26E-06	-0.002	0.002	0.02%	1	-0.01	-0.018	-3.78E-04	99.98%	0.04	-0.01	-0.019	-2.01E-04	0.05
Physiological arousal - Bravery - Perceived importance	-4.29E-04	-0.003	0.002	3.52%	0.74	-0.012	-0.025	0.001	96.48%	0.07	-0.012	-0.025	9.05E-04	0.07
Physiological arousal - Perseverance - Perceived importance	-3.22E-04	-0.003	0.002	2.65%	0.82	-0.012	-0.025	9.42E-04	97.35%	0.07	-0.012	-0.025	9.05E-04	0.07
Physiological arousal - Zest - Perceived importance	5.37E-04	-0.002	0.003	4.05%	0.69	-0.013	-0.026	1.19E-04	95.95%	0.05	-0.012	-0.025	9.05E-04	0.07
Physiological arousal - Courage - Perceived importance	-1.35E-04	-0.002	0.003	1.11%	0.92	-0.012	-0.025	8.00E-04	98.89%	0.07	-0.012	-0.025	9.05E-04	0.07
Dental fear - Perseverance - Perceived importance	2.41E-04	-5.43E-04	0.001	6.00%	0.55	-0.004	-0.007	-5.29 e-4	94.00%	0.02	-0.004	-0.007	-2.07 e-4	.037
